# An update on the roles of circular RNAs in osteosarcoma

**DOI:** 10.1111/cpr.12936

**Published:** 2020-10-25

**Authors:** Zheng Li, Xingye Li, Derong Xu, Xin Chen, Shugang Li, Lin Zhang, Matthew T. V. Chan, William K. K. Wu

**Affiliations:** ^1^ Department of Orthopaedic Surgery Peking Union Medical College Hospital Chinese Academy of Medical Sciences and Peking Union Medical College Beijing China; ^2^ Department of Orthopedic Surgery Beijing Jishuitan Hospital Fourth Clinical College of Peking University Jishuitan Orthopaedic College of Tsinghua University Beijing China; ^3^ Department of Orthopedics The Affiliated Hospital of Qingdao University Qingdao China; ^4^ Department of Anaesthesia and Intensive Care Peter Hung Pain Research Institute The Chinese University of Hong Kong Hong Kong City Hong Kong; ^5^ State Key Laboratory of Digestive Diseases Centre for Gut Microbiota Research Institute of Digestive Diseases and LKS Institute of Health Sciences The Chinese University of Hong Kong Hong Kong City Hong Kong

**Keywords:** CircRNAs, CircTCF25, ncRNAs, osteosarcoma

## Abstract

Osteosarcoma is the most common primary bone malignancy and is a neoplasm thought to be derived from the bone‐forming mesenchymal stem cells. Aberrant activation of oncogenes and inactivation of tumour suppressor genes by somatic mutations and epigenetic mechanisms play a pivotal pathogenic role in osteosarcoma. Aside from alterations in these protein‐coding genes, it has now been realized that dysregulation of non‐coding RNAs (ncRNAs), including microRNAs (miRNAs), long non‐coding RNAs (lncRNAs) and the recently discovered circular RNAs (circRNAs), is crucial to the initiation and progression of osteosarcoma. CircRNAs are single‐stranded RNAs that form covalently closed loops and function as an important regulatory element of the genome through multiple machineries. Recently, an increasing number of studies suggested that circRNAs also played critical roles in osteosarcoma. This review summarizes recent development and progression in circRNA transcriptome analysis and their functions in the modulation of osteosarcoma progression.

## Graphical abstract

The representative diagram of circRNA mediated ceRNA network and oncogene signaling pathways. The current study in osteosarcoma highlights the regulatory relationship of the circRNA‐miRNA‐mRNA network for different signaling pathways. Multiple identified circRNAs function as miRNA sponge and subsequently upregulate relevant target genes expression level.

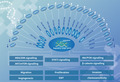

## 1 INTRODUCTION

**Figure 1:**
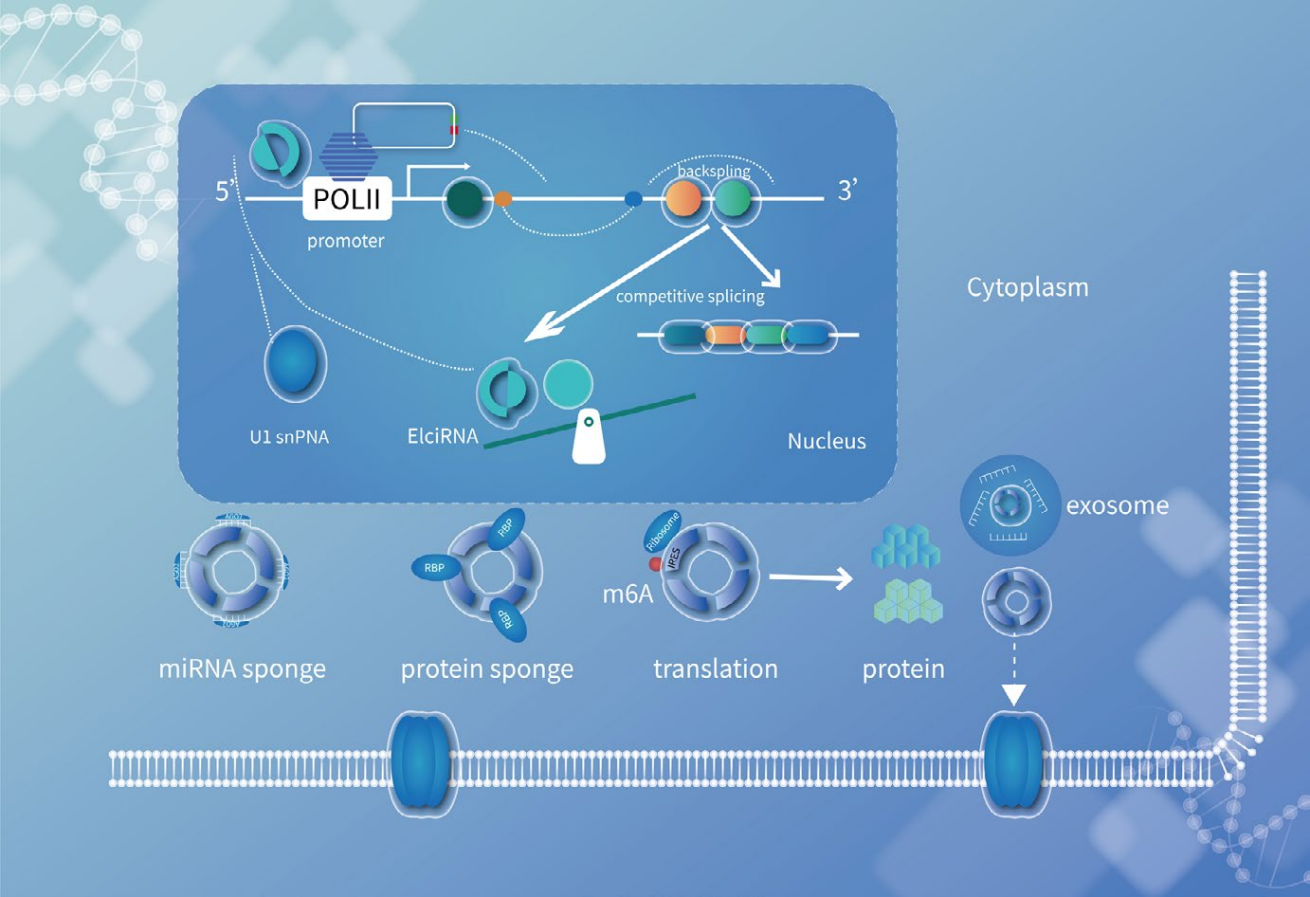
Potential function mechanisms of circRNAs. CircRNA can play as miRNA sponges and subsequently modulate its target gene expression. CircRNA can bind to several proteins to mediate their actions. Abundant m6A modification and IRES can promote a lot of circRNAs to translation

Osteosarcoma, a neoplasm thought to be derived from the bone‐forming mesenchymal stem cells, is the most common primary bone malignancy, predominantly involving metaphyseal regions of the long bones (eg, proximal end of tibia or humerus and distal end of femur) which are the most rapidly growing parts of the skeleton in children and adolescents.^1^, ^2^, ^3^, ^4^, ^5^ The incidence of osteosarcoma follows a bimodal age distribution with ages between 10 and 30 primarily affected.^6^, ^7^, ^8^, ^9^, ^10^ The second peak appears in the elderly in which osteosarcoma very often comes as a later cancer secondary to radiation exposure or is associated with Paget disease (a disorder of bone remodelling resulting in structural weakening).^11^, ^12^, ^13^, ^14^, ^15^, ^16^ With the advent of neoadjuvant and adjuvant chemotherapy with cisplatin, doxorubicin and high‐dose methotrexate and the advances in surgery, the 5‐year survival rate of patients with osteosarcoma has improved from ~20% before the 1980s to currently ~ 70%.^17^, ^18^, ^19^, ^20^, ^21^, ^22^ Nevertheless, half of the patients still do not survive for longer than 10 year.^23^, ^24^, ^25^ Thus, it is important to identify new therapeutic targets for tackling this devastating and potentially fatal disease (Figure 1).

Aberrant activation of oncogenes (eg, *COPS3*, *PIK3CA*, *CARD11*, *MYC*) and inactivation of tumour suppressor genes (eg, *TP53*, *RB1*, *ATRX*, *DLG2*, *BRCA1*/*2*) by somatic mutations and epigenetic mechanisms play a pivotal pathogenic role in osteosarcoma.^26^, ^27^, ^28^, ^29^, ^30^, ^31^, ^32^, ^33^, ^34^, ^35^, ^36^, ^37^ Aside from alterations in these protein‐coding genes, it has now been realized that dysregulation of non‐coding RNAs (ncRNAs), including microRNAs (miRNAs), long non‐coding RNAs (lncRNAs) and the recently discovered circular RNAs (circRNAs), is crucial to the initiation and progression of osteosarcoma.^2^, ^38^, ^39^, ^40^, ^41^, ^42^, ^43^, ^44^, ^45^, ^46^ CircRNAs are single‐stranded RNAs that form covalently closed loops and function as an important regulatory element of the genome through multiple machineries, including transcription regulation, modulation of alternative splicing, sponging of miRNAs and direct interactions with RNA‐binding proteins.^47^, ^48^, ^49^, ^50^, ^51^, ^52^ A subset of circRNAs also retains protein‐coding capacity and was found to be translated in vivo.^53^, ^54^, ^55^

A growing body of evidence now indicates that circRNA expression is dysregulated in most, if not all, types of cancer, including glioma, gastric cancer, bladder cancer, hepatocellular carcinoma, lung cancer, gallbladder carcinoma and renal cell carcinoma, in which circRNA deregulation perturbs cancer‐related phenotypes, such as cell survival, proliferation, differentiation, metabolism and invasion/metastasis.^49^, ^51^, ^52^, ^54^, ^56^, ^57^, ^58^, ^59^, ^60^, ^61^, ^62^ Recently, an increasing number of studies suggested that circRNAs also played critical roles in osteosarcoma.^63^, ^64^, ^65^ For instance, circLRP6 was shown to promote osteosarcoma progression through inhibiting APC and KLF2 expression.^66^ Zhao et al^67^ also reported that circSAMD4A increased osteosarcoma growth by sponging miR‐1244 and thereby derepressing MDM2 expression. Previously, Zhang et al^68^ elegantly summarized the roles of circRNAs in osteosarcoma. As circRNA research is a rapidly evolving field, over a dozen of original articles related to circRNAs in osteosarcoma have been published since then. It is therefore timely to update the recent progress.

In this review, we first summarize circRNA profiling studies on osteosarcoma so as to provide a comprehensive curation of available datasets which may facilitate subsequent studies, followed by in‐depth discussion of functions and mechanisms of action of newly discovered osteosarcoma‐related circRNAs not covered by the review of Zhang et al.^68^ The potential diagnostic, prognostic and therapeutic utilities of circRNAs in the clinical management of osteosarcoma are also addressed.

## 2 circRNA EXPRESSION PROFILING AND INTEGRATIVE ANALYSIS IN OSTEOSARCOMA

Profiling circRNA expression using microarray or whole‐transcriptome sequencing followed by validation with reverse transcription‐quantitative PCR (RT‐qPCR) is the most frequent method to identify and confirm deregulated expression of circRNAs in specific disease states.^69^, ^70^, ^71^, ^72^ In this connection, attempts have been made to profile circRNAs specific to osteosarcoma or related to its resistance to chemotherapy.

Liu et al^65^ extracted total RNA from the human osteosarcoma cell lines U2OS, MG63, HOS and 143B and the human normal osteoblast hFOB1.19, and then subjected the RNA samples to digestion with RNase R to remove linear RNAs. The enriched circRNAs were then amplified and transcribed into fluorescent complementary RNA for hybridization onto a human circRNA array. A total of 252 circRNAs were found to be differentially expressed between the human osteosarcoma cell lines and hFOB1.19 (71 upregulated and 181 downregulated in osteosarcoma cell lines). Nevertheless, among the 12 selected differentially expressed circRNAs, only the upregulation of circRNA_103801 and the downregulation of circRNA_104980 were successfully validated in both osteosarcoma cell lines and human osteosarcoma tissues by RT‐qPCR, suggesting a low validity of using cell line models to identify osteosarcoma‐specific circRNAs. For the validated circRNA_103801, its expression was found to be strongly correlated with TANC1 (tetratricopeptide repeat, ankyrin repeat and coiled‐coil containing 1), an oncogene previously reported in rhabdomyosarcoma. In another study, Xi et al^73^ used the Illumina HiSeq platform to profile circRNA expression in three pairs of osteosarcoma and para‐cancerous tissues. A total of 259 circRNAs were found to be differentially expressed (32 upregulated and 127 downregulated in osteosarcoma). Among the five selected differentially expressed circRNAs identified by RNA sequencing, significant upregulation of circ_2137 and circ_20403 and downregulation of circ_32279 and circ_24831 were successfully verified in 10 independent pairs of osteosarcoma and para‐cancerous tissues, suggesting an overall high validity of the sequencing findings. Pathway enrichment analysis of genes hosting the differentially expressed circRNAs further indicated the potential involvement of phosphatidylinositol signalling, an oncogenic pathway frequently engaged in tumorigenesis. A recent study used PCR array to profile circRNAs in 30 frozen samples of osteosarcoma and their corresponding adjacent tissues.^74^ The authors identified 10 differentially expressed circRNAs (six upregulated and four downregulated in osteosarcoma) with circ_001621 as the top upregulated circRNA.

Qiu et al^75^ performed an integrative analysis of publicly available gene expression datasets on osteosarcoma downloaded from the Gene Expression Omnibus (GEO) database. They identified 15 downregulated circRNAs, 136 upregulated miRNAs and 52 downregulated mRNAs in osteosarcoma, among which 14 circRNAs, 24 miRNAs and 52 mRNAs formed a circRNA‐miRNA‐mRNA network. Pathway analysis revealed these mRNAs were enriched in ‘phosphoinositide 3‐kinase‐Akt signalling pathway’, ‘proteoglycans in cancer’, ‘cell cycle’ and ‘FoxO signalling pathway’. Importantly, low expression of four mRNAs (ARID5B, ELL2, PHC2 and STAT4) in the circRNA‐regulated network was associated with significantly shortened overall survival of osteosarcoma patients.

Aside from osteosarcoma‐specific circRNAs, efforts have been made to identify circRNAs pertinent to chemosensitivity in osteosarcoma. Zhu et al compared the circRNA expression profiles of three chemoresistant osteosarcoma cell lines (MG63/DXR, U2OS/DXR, KHOS/DXR) that were insensitive to cisplatin, doxorubicin and methotrexate with their cognate chemosensitive osteosarcoma cell lines (MG63, U2OS, KHOS) using next‐generation RNA sequencing.^76^ A total of 80 circRNAs were found to be differentially expressed (57 upregulated and 23 downregulated in chemoresistant cell lines). Validation with RT‐qPCR indicated that nine out of 10 selected circRNAs showed consistent direction of deregulation as shown by RNA sequencing. Pathway analyses of the differentially expressed circRNAs and their parental genes revealed biological processes pertinent to chemoresistance, including glycolysis/gluconeogenesis, ABC transporters, and vascular endothelial growth factor signalling (Table 1).

**Table 1:** circRNAs expression profiles in osteosarcoma

Num	Method	Sample	Upregulated	Downregulated	Reference
1	Microarray RT‐PCR	Osteosarcoma cell lines and hFOB1.19	71 LncRNAs circRNA_103801	181 LncRNAs circRNA_104980	65
2	Microarray RT‐PCR	Osteosarcoma tissues and para‐cancerous tissues	32 LncRNAs circ_2137 circ_20403	127 LncRNAs circ_32279 circ_24831	73
3	Microarray RT‐PCR	Osteosarcoma tissues and para‐cancerous tissues	6 lncRNAs circ_001621	4 lncRNAs	74
4	Bioinformatics	Gene expression datasets	15 lncRNAs		75
5	Microarray RT‐PCR	Chemoresistant and chemosensitive osteosarcoma cell line	57 lncRNAs	23 lncRNAs X	76

## 3 FUNCTIONS AND MECHANISMS OF ACTION OF NEWLY DISCOVERED circRNAs IN OSTEOSARCOMA

### 3.1 Oncogenic circRNAs

#### 3.1.1 CircTCF25

circTCF25 was first identified as an oncogenic circRNA in bladder carcinoma.^77^ Wang et al reported that the expression of circTCF25 was significantly higher in osteosarcoma tissues as compared with morphologically normal bone tissues from the same patients. Functionally, enforced expression of circTCF25 promoted osteosarcoma cell proliferation, migration and invasion, accompanied by corresponding alterations of phenotype‐related proteins (upregulation of cyclin D1 and CDK6 for increased cell proliferation; upregulation of MMP2, MMP9 and Vimentin and downregulation of TIMP‐1 for enhanced cell migration and invasion). Mechanistically, overexpression of circTCF25 reduced the levels of miR‐206 in osteosarcoma cells, where transfection of miR‐206 mimic abrogated circTCF25‐induced pro‐tumorigenic phenotypes and the associated protein expression. In this regard, the MEK/ERK and AKT/mTOR pathways were found to be the downstream pathways inhibited by miR‐206.^78^

#### 3.1.2 CircPVT1

CircPVT1 is a circRNA derived from the genomic region that also encodes the oncogenic lncRNA PVT1.^79^ Liu et al^80^ found that circPVT1 increased the invasiveness and metastatic capacity of osteosarcoma cells in vitro via promoting epithelial‐mesenchymal transition through sponging miR‐205‐5p and thereby derepressing c‐FLIP. Yan et al^81^ also showed that circPVT1 played as one oncogene in development of osteosarcoma. These studies suggested that circPVT1 is an oncogenic circRNA in osteosarcoma, where it contributes to metastasis through upregulating c‐FLIP.

#### 3.1.3 CircMMP9

CircMMP9 has been shown to promote glioblastoma.^82^ Pan et al^83^ found that the expression of circMMP9 was higher in osteosarcoma tissues, where high expression was associated with larger tumours and more advanced TNM staging. The overall survival of osteosarcoma patients with high tumoral expression of circMMP9 was also shorter than those with low circMMP9 expression. Functionally, knockdown of circMMP9 reduced the viability, colony‐forming ability, migration and invasion of osteosarcoma cells, suggesting an oncogenic role for circMMP9. The pro‐tumorigenic actions of circMMP9 were found to be mediated through sponging of miR‐1265 and the subsequent depression of CHI3L1 (chitinase‐3‐like protein 1).^83^

#### 3.1.4 Circ_001621

Circ_001621 was identified as the top upregulated circRNA in osteosarcoma by PCR array, and its high expression was associated with more advanced TNM staging and shortened overall survival.^74^ In this regard, miR‐578 was found to be the target of circ_001621 in osteosarcoma where their expression exhibited strong negative correlation. Functional experiments and mechanistic studies revealed that circ_001621 promoted the proliferation and migration of osteosarcoma cells in vitro via attenuating miR‐558‐mediated repressing of VEGF and the downstream expression of CDK4 and MMP9. Importantly, circ_001621 enhanced the metastatic capacity of osteosarcoma to the lung and liver in vivo accompanied by the upregulating of VEGF, CDK4 and MMP9.^74^ Collectively, circ_001621 is an oncogenic circRNA promoting the metastasis of osteosarcoma by regulating the miR‐558‐VEGF pathway.

#### 3.1.5 Circ‐0000285

Circ‐0000285 is another circRNA generated from *HIPK3*. Zhang et al found that overexpression of circ‐0000285 increased the colony‐forming ability, proliferation and migration of osteosarcoma cells in vitro and accelerated the growth of osteosarcoma xenografts in vivo whereas knockdown of circ‐0000285 produced the opposite effects. Mechanistic investigations suggested that circ‐0000285 bound to miR‐599 to derepress TGF‐β_2_ (transforming growth factor‐beta 2).^84^ In this connection, TGF‐β signalling is known to contribute to osteosarcoma progression by promoting angiogenesis, bone remodelling, cell migration and immune evasion.^85^

#### 3.1.6 Circ_0001658

Circ_0001658 was found to be upregulated in osteosarcoma tissues as compared with normal bone tissues. Functional experiments suggested that circ_0001658 promoted the proliferation, migration, and invasion of osteosarcoma cells and impeded apoptosis. These pro‐tumorigenic effects were mediated by sponging miR‐382‐5p and the subsequent depression of YB‐1. Taken together, circ_0001658 is an oncogenic circRNA via modulating the miR‐382‐5p/YB‐1 axis.^86^

#### 3.1.7 Circ_0102049

The expression of circ_0102049 was found to be higher in osteosarcoma tissue samples as compared with their paired non‐cancerous tissues.^87^ In patients with osteosarcoma, high tumoral expression of circ_0102049 was associated with larger tumour size, lung metastasis and shortened overall survival. Gain‐of‐function and loss‐of‐function experiments indicated that circ_0102049 acted as an oncogene by promoting osteosarcoma cell proliferation, migration and invasion and inhibiting apoptosis. Mechanistically, circ_0102049 was found to upregulate MDM2 (an E3 ubiquitin‐protein ligase responsible degradation of p53) through sponging miR‐1304‐5p. To this end, knockdown of MDM2 attenuated the stimulatory effects of circ_0102049 overexpression on osteosarcoma cell viability, migration and invasion,^87^ suggesting the miR‐1304‐5p/MDM2 axis is an effector pathway for the oncogenic action of circ_0102049.

#### 3.1.8 CircEPSTI1

Tan et al^88^ reported that circEPSTI1 was significantly upregulated in osteosarcoma where knockdown of this circRNA suppressed cell proliferation and migration, suggesting an oncogenic role. Mechanistically, circEPSTI1 upregulated the expression of MCL1 (myeloid cell leukaemia 1) via reducing the availability of miR‐892b.^88^ These data suggested an important role for circEPSTI1‐miR‐892b‐MCL1 axis in the progression of osteosarcoma.

#### 3.1.9 Circ‐0060428

Cao and Liu reported that Circ‐0060428 was expressed at significantly higher levels in osteosarcoma cell lines (U2OS, 143B, SAOS‐2, HOS) as compared with the human normal osteoblast hFOB1.19.^89^ Functionally, knockdown of circ‐0060428 induced apoptosis of U2OS and HOS osteosarcoma cells, suggesting this circRNA functioned as an oncogenic circRNA. Mechanistic studies found that circ‐0060428 bound to miR‐375 and thereby depressing RBPJ (a transcription factor positively regulating Notch signalling). In this connection, miR‐375 inhibitor nullified the pro‐apoptotic effect of circ‐0060428 knockdown.^89^ These findings suggest that upregulation of circ‐0060428 could confer apoptosis resistance to osteosarcoma cells by sponging miR‐375. Nevertheless, whether RBPJ is functionally involved in the oncogenic action of circ‐0060428 remains unclear. Moreover, the upregulation of circ‐0060428 needs to be confirmed in human osteosarcoma tissues.

#### 3.1.10 Circ_ANKIB1

Du et al^90^ illustrated that circ_ANKIB1 directly sponged miR‐19b in the osteosarcoma cell and Circ_ANKIB1 enhanced expression of miR‐19b and suppressed SOCS3 expression, which is its downstream gene. Knockdown of miR‐19b or circ_ANKIB1 suppressed cell invasion and growth and induced cell apoptosis via modulating STAT3 pathway. These data indicated that circ_ANKIB1 acted as one oncogene and potential treatment target for osteosarcoma.

#### 3.1.11 CircMYO10

Chen et al^91^ illustrated that miR‐370‐3p was decreased and circMYO10 was overexpressed in osteosarcoma samples and cells. Inhibition expression of circMYO10 suppressed EMT development and growth through regulating miR‐370‐3p/RUVBL1/β‐catenin/LEF1 axis. These results revealed that circMYO10 played as one oncogene role in progression of osteosarcoma.

#### 3.1.12 CircLRP6

Previous study noted that circLRP6 level was overexpressed in osteosarcoma samples and was correlated with poor overall survival and shorter disease‐free survival.^92^ Knockdown of circLRP6 inhibited cell migration, invasion and growth and induced cell cycle arrested and apoptosis. The interaction between EZH2 and LSD1 with circLRP6 regulates their binding to promoter regions of APC and KLF2. Inhibition expression of circLRP6 decreased binding abilities of EZH2, LSD1 to APC, KLF2. Furthermore, the oncogene effect of circLRP6 on osteosarcoma can be reversed with APC. These data indicated that circLRP6 served as one oncogene through binding to EZH2 and LSD1 to suppress APC and KLF2 expression.

#### 3.1.13 CircSAMD4A

Zhao et al^67^ noted that circSAMD4 was upregulated in osteosarcoma specimens compared with adjacent non‐cancerous specimens. Ectopic expression of circSAMD4A induced osteosarcoma cell growth in vitro and in vivo and increased cells stemness characteristics. Moreover, they illustrated that circSAMD4A sponged miR‐1244 and identified MDM2 was one direct target gene of miR‐1244. Their data illustrated that miR‐1244/MDM2/ circSAMD4A modulator loop may be one treatment target for osteosarcoma.

#### 3.1.14 Circ‐0001785

Previous reference noted that circ‐0001785 was overexpressed in cell lines of osteosarcoma.^93^ Downregulation expression of circ‐0001785 inhibited cell growth and enhanced osteosarcoma cell apoptosis. In addition, they showed that circ‐0001785 can sponge miR‐1200 expression and suppress HOXB2 expression, which is one target gene of miR‐1200. They showed that circ‐0001785 modulated Bcl‐2 pathway and Akt/PI3K signalling in the osteosarcoma. These results suggested that circ‐0001785 modulates osteosarcoma pathogenesis via regulating miR‐1200 to enhance HOXB2 expression.

#### 3.1.15 Circ_ORC2

Li et al^94^ illustrated that circ_ORC2 was distributed in cell cytoplasm and overexpressed in several cell lines of osteosarcoma. Circ_ORC2 can sponge miR‐19a expression, and knockdown of circ_ORC2 suppressed miR‐19a and enhanced its target gene PTEN expression. Knockdown of circ_ORC2 inhibited cell invasion and growth and enhanced cell apoptosis. Thus, they indicated that circ_ORC2 binds with the miR‐19a and increased its expression, then enhancing Akt pathway and suppressing its downstream gene PTEN expression to induce cell invasion and growth. These data provided a new oncogene circ_ORC2 for osteosarcoma.

#### 3.1.16 CircRNA_100876

Jin et al^95^ showed that circRNA_100876 level was overexpressed in osteosarcoma tissues and was associated with differentiation degree and size of tumour. CircRNA_100876 knockdown suppressed tumour growth in vivo and in vitro. Inhibition expression of circRNA_100876 suppressed cell growth and induced cell apoptosis and decreased cell cycle. Moreover, the expression of circRNA_100876 was negatively correlated with miR‐136 expression. Knockdown of miR‐136 can reverse the inhibition of cell growth induced with silencing circRNA_100876. These data indicate that circRNA_100876 may act as one promising treatment target and biomarker for osteosarcoma.

#### 3.1.17 CircTADA2A

Wu et al^96^ revealed that circTADA2A was overexpressed in osteosarcoma cells and samples and inhibition of circTADA2A suppressed tumour metastasis and tumorigenesis in vivo and inhibited cell invasion, growth and migration in vitro. Moreover, they noted that circTADA2A sponged miR‐203a‐3p expression and enhanced CREB3 expression, which was found as one driver gene of osteosarcoma. CREB3 overexpression or knockdown of miR‐203a‐3p can reverse circTADA2A silencing‐leading impairment of tumour behaviour. These results suggested that circTADA2A acted as one oncogene through regulating miR‐203a‐3p/CREB3 axis in osteosarcoma.

#### 3.1.18 Circ_0000885

Zhu et al^97^ indicated that circ_0000885 expression was upregulated in serum samples and tissue from osteosarcoma patients and higher expression of circ_0000885 was correlated with Enneking stage. Cases with high tumour and serum levels of the circ_0000885 had the lower rates of overall survival and disease‐free survival. These results suggested that circ_0000885 may act as one diagnostic biomarker for the osteosarcoma.

### 3.2 Tumour‐suppressing circRNAs

#### 3.2.1 Circ0021347

The expression of circ0021347 was lower in human osteosarcoma tissues and cell lines than their normal counterparts. In patients with osteosarcoma, the low expression of circ0021347 was associated with more advanced tumour‐node‐metastasis (TNM) staging and shortened overall survival, suggesting circ0021347 functions as a tumour suppressor.^98^ Nevertheless, the mechanism of action of circ0021347 is presently unclear.

#### 3.2.2 Circ_0000190

Circ_0000190 has been shown to inhibit the progression of multiple myeloma through sponging miR‐767‐5p. Li et al reported that circ_0000190 was expressed at significantly lower levels in human osteosarcoma tissues and cell lines as compared with para‐cancerous normal tissues and hFOB1.19 normal osteoblasts, respectively.^99^ Enforced expression of circ_0000190 suppressed osteosarcoma cell proliferation, migration and invasion. Mechanistically, circ_0000190 bound to miR‐767‐5p and thereby derepressing the downstream target TET1. The levels of circ_0000190 in extracellular nanovesicles isolated from plasma of osteosarcoma patients were also significantly lower than those from healthy subjects, suggesting the potential diagnostic use of circulating circ_0000190. In this regard, low abundance of circ_0000190 in extracellular nanovesicles could distinguish cases from controls with an AUROC of 0.8894.^99^

#### 3.2.3 Circ‐LARP4

Hu et al^100^ showed that circ‐LARP4 level was decreased in osteosarcoma tissues compared to non‐tumour samples and circ‐LARP4 was associated with Enneking stage. Patients with high circ‐LARP4 level indicated enhanced tumour cell necrosis rates to chemotherapy after resection. The higher expression of circ‐LARP4 was associated with prolonged overall survival and disease‐free survival. Ectopic expression of circ‐LARP4 enhanced MG63 cells chemosensitivity to doxorubicin and cisplatin but not the methotrexate. Moreover, overexpression of miR‐424 decreased the chemosensitivity effect in circ‐LARP4 overexpression handled MG63 cells. Their data suggested that high expression of circ‐LARP4 was associated with prolonged survival profiles and decreased Enneking stage and overexpression of circ‐LARP4 enhanced chemosensitivity to doxorubicin and cisplatin through sponging miR‐424 in development of osteosarcoma.

### 3.3 Circ‐ITCH, a circRNA with an ambiguous role

**Figure 2:**
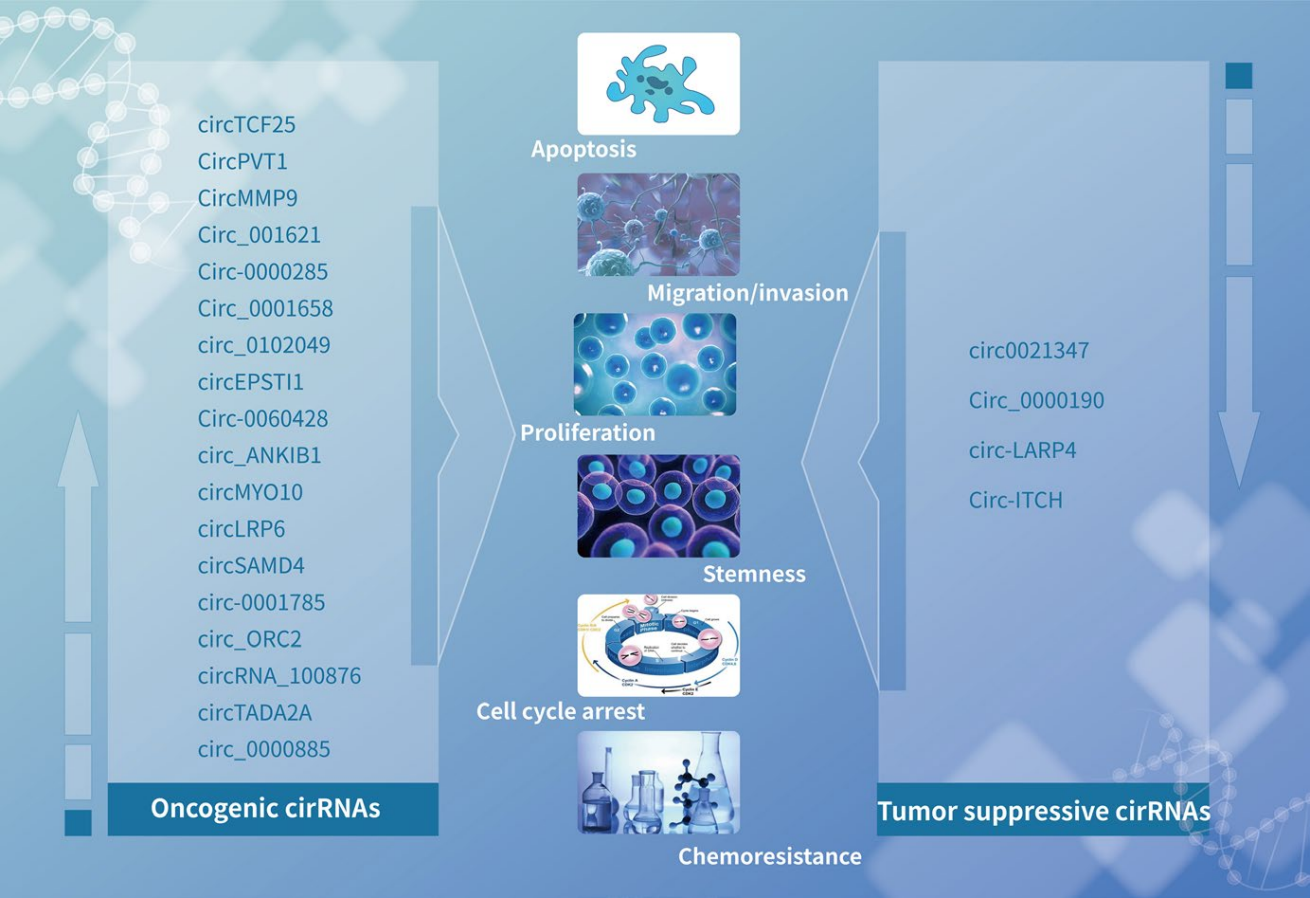
Oncogenic and tumour suppressor circRNAs in osteosarcoma and their influence on biological processes, including proliferation, apoptosis, chemoresistance, migration/invasion, cell cycle arrest and stemness. The left panel shows oncogenic circRNAs with upregulated expression, while the right panel indicates tumour‐suppressive circRNAs with downregulated expression in osteosarcoma

**Figure 3:**
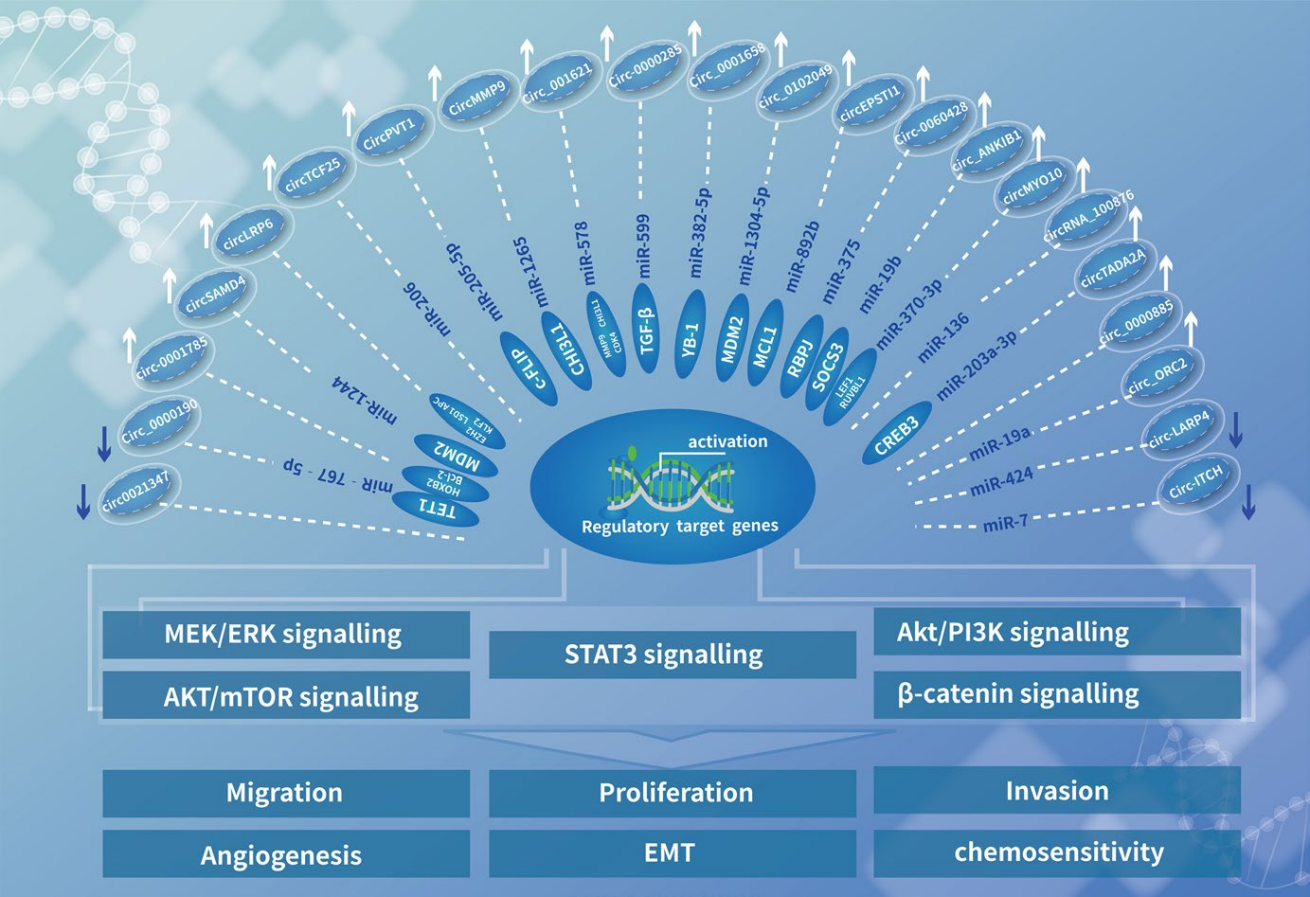
The representative diagram of circRNA mediated ceRNA network and oncogene signalling pathways. The current study in osteosarcoma highlights the regulatory relationship of the circRNA‐miRNA‐mRNA network for different signalling pathways. Multiple identified circRNAs function as miRNA sponge and subsequently upregulate relevant target genes expression level

Circ‐ITCH was found to act as a tumour‐suppressing circRNA in oesophageal squamous cell carcinoma,^101^ colorectal cancer^102^ and lung cancer.^103^ In contrast with its inhibitory role in these cancer types, Li et al^104^ reported that cir‐ITCH was expressed at significantly higher levels in a panel of osteosarcoma cell lines as compared with hFOB1.19 normal osteoblasts. Importantly, enforced expression of cir‐ITCH promoted osteosarcoma cell proliferation, migration and invasion whereas knockdown of cir‐ITCH produced the opposite effects. The oncogenic action of cir‐ITCH was mediated by the sponging of miR‐7 and the subsequent enhancement of epidermal growth factor receptor (EGFR) expression and activation the downstream MEK‐ERK cascade. To this end, erlotinib (an EGFR tyrosine kinase inhibitor) nullified the pro‐migratory and pro‐invasive effects of cir‐ITCH.^104^ Ren et al^105^ also found that circ‐ITCH played asa tumour suppressor in progression of osteosarcoma by modulating miR‐22 (Tables 2 and 3; Figures 2 and 3).

**Table 2:** Dysregulated circRNAs in osteosarcoma

Name	Dysregulation	Sponge target	Function	Related gene	Role	Reference
circTCF25	Upregulated	miR‐206	Proliferation, migration and invasion	MEK/ERK AKT/mTOR	Oncogenic	78
CircPVT1	Upregulated	miR‐205‐5p miR‐526b	Invasion EMT	c‐FLIP FOXC2	Oncogenic	80, 81
CircMMP9	Upregulated	miR‐1265	Proliferation, colony, migration, invasion	CHI3L1	Oncogenic	83
Circ_001621	Upregulated	miR‐578	Proliferation migration	VEGF CDK4 MMP9	Oncogenic	74
Circ‐0000285	Upregulated	miR‐599	Colony proliferation migration	TGF‐β	Oncogenic	84
Circ_0001658	Upregulated	miR‐382‐5p	Proliferation migration invasion	YB‐1	Oncogenic	86
circ_0102049	Upregulated	miR‐1304‐5p	Proliferation migration invasion apoptosis	MDM2	Oncogenic	87
circEPSTI1	Upregulated	miR‐892b	Proliferation migration	MCL1	Oncogenic	88
Circ‐0060428	Upregulated	miR‐375	Apoptosis	RBPJ	Oncogenic	89
circ_ANKIB1	Upregulated	miR‐19b	Invasion growth apoptosis	SOCS3 STAT3	Oncogenic	90
circMYO10	Upregulated	miR‐370‐3p	EMT growth	RUVBL1 β‐catenin LEF1	Oncogenic	91
circLRP6	Upregulated		Migration invasion growth cell cycle apoptosis	EZH2 LSD1 APC KLF2	Oncogenic	92
circSAMD4	Upregulated	miR‐1244	Growth stemness	MDM2	Oncogenic	67
circ‐0001785	Upregulated	miR‐1200	Growth apoptosis	Akt/PI3K Bcl‐2 HOXB2	Oncogenic	
circ_ORC2	Upregulated	miR‐19a	Invasion growth apoptosis	PTEN Akt	Oncogenic	94
circRNA_100876	Upregulated	miR‐136	Growth apoptosis cycle		Oncogenic	95
circTADA2A	Upregulated	miR‐203a‐3p	Metastasis invasion growth migration	CREB3	Oncogenic	96
circ_0000885	Upregulated				Oncogenic	97
circ0021347	Downregulated				Tumour suppressor	98
Circ_0000190	Downregulated	miR‐767‐5p	Proliferation migration invasion	TET1	Tumour suppressor	99
circ‐LARP4	Downregulated	miR‐424	Chemosensitivity		Tumour suppressor	101
Circ‐ITCH	Downregulated	miR‐7 miR‐2	Proliferation migration invasion	MEK ERK	Tumour suppressor	104, 105

**Table 3:** CircRNAs as diagnostic and prognostic biomarkers in Osteosarcoma

Name	Deregulation	Function	Clinicopathological association	Reference
circMMP9	Upregulated	Independent prognostic indicator	Larger tumours advanced TNM staging overall survival	83
Circ_001621	Upregulated	Independent prognostic indicator	TNM staging overall survival	74
circ_0102049	Upregulated	Independent prognostic indicator	Tumour size lung metastasis overall survival	87
circLRP6	Upregulated	Independent prognostic indicator	Overall survival disease‐free survival	92
circRNA_100876	Upregulated	Independent prognostic indicator	Differentiation degree tumour size	95
circ_0000885	Upregulated	Independent prognostic Indicator and Diagnosis biomarker	Enneking stage overall survival disease‐free survival	97
circ0021347	Downregulated	Independent prognostic indicator and diagnosis biomarker	Advanced TNM staging overall survival	98
circ‐LARP4	Downregulated	Independent prognostic indicator and diagnosis biomarker	Enneking stage overall survival and disease‐free survival	101

## 4 CONCLUSIONS AND FUTURE PERSPECTIVES

Osteosarcoma is one multi‐factor and multi‐step comprehensive disease, pathogenesis mechanism is still unclear, and more efforts are in urgent need. CircRNAs function as an important regulatory element of the genome via several machineries, including transcription regulation, modulation of alternative splicing, sponging of miRNAs and direct interactions with RNA‐binding proteins. CircRNAs may play as tumour suppressors or activators in tumour and have been applied in modulating cancer metastasis and chemoresistance. As summarized in our review, circRNAs are acting critical roles in many biological processes in the procedures in the progression and development of osteosarcoma including cell apoptosis, invasion, growth, differentiation, migration, drug resistance and cachexia. As one crucial biomarker for prognosis and diagnosis of osteosarcoma, circRNA is one potential target for therapy of osteosarcoma. This use of circRNAs as therapeutics has vast tremendously and may supply a new method to treat osteosarcoma.

Here are some future suggestions for circRNAs study. Firstly, more exact mechanisms about how circRNAs participated in progression of osteosarcoma need to further research, potentially via using online databases. Secondly, further study on circRNAs in osteosarcoma needs to use non‐invasive specimens such as saliva, urine and blood. Thirdly, since the purpose of circRNA study is to use circRNAs for clinical application for osteosarcoma, generous clinical markers experiments and cell function mechanism studies are required in future.

## CONFLICT OF INTEREST

The authors declare that they have no competing interests.

## AUTHORS’ CONTRIBUTIONS

ZL, XYL, DRX, XC and SGL collected the related paper. ZL, XYL and DRX drafted and wrote the manuscript. LZ, MTVC and WKKW revised the manuscript. SGL participated in the design of the review and helped to draft and revise the manuscript. All authors read and approved the final manuscript.

## Data Availability

Research data are not shared.

## References

[1] Li Z , Yu X , Shen JX . Long non‐coding RNAs: emerging players in osteosarcoma. Tumor Biol. 2016;37(3):2811‐2816. 10.1007/s13277-015-4749-4 26718212

[2] Li Z , Shen JX , Chan MTV , Wu WKK . MicroRNA‐379 suppresses osteosarcoma progression by targeting PDK1. J Cell Mol Med. 2017;21(2):315‐323. 10.1111/jcmm.12966 27781416 PMC5264134

[3] Zhao J , Gao Z , Zhang C , Wu H , Gu R , Jiang R . Long non‐coding RNA ASBEL promotes osteosarcoma cell proliferation, migration and invasion by regulating microRNA‐21. J Cell Biochem. 2018;119(8):6461‐6469. 10.1002/jcb.26671 29323740

[4] Yang C , Wu K , Wang S , Wei G . Long non‐coding RNA XIST promotes osteosarcoma progression by targeting YAP via miR‐195‐5p. J Cell Biochem. 2018;119(7):5646‐5656. 10.1002/jcb.26743 29384226

[5] Xu RD , Feng F , Yu XS , Liu ZD , Lao LF . LncRNA SNHG4 promotes tumour growth by sponging miR‐224‐3p and predicts poor survival and recurrence in human osteosarcoma. Cell Prolif. 2018;51(6): 10.1111/cpr.12515 PMC6528889 30152090

[6] Ba ZW , Gu LL , Hao SN , Wang XF , Cheng ZP , Nie GC . Downregulation of lncRNA CASC2 facilitates osteosarcoma growth and invasion through miR‐181a. Cell Prolif. 2018;51(1): 10.1111/cpr.12409 PMC6528952 29194827

[7] Duan Z , Choy E , Harmon D , et al. MicroRNA‐199a‐3p is downregulated in human osteosarcoma and regulates cell proliferation and migration. Mol Cancer Ther. 2011;10(8):1337‐1345. 10.1158/1535-7163.MCT-11-0096 21666078 PMC3711153

[8] Zhao G , Cai C , Yang T , et al. MicroRNA‐221 induces cell survival and cisplatin resistance through PI3K/Akt pathway in human osteosarcoma. PLoS One. 2013;8(1):e53906 10.1371/journal.pone.0053906 23372675 PMC3553141

[9] Sun XH , Geng XL , Zhang J , Zhang C . miRNA‐646 suppresses osteosarcoma cell metastasis by downregulating fibroblast growth factor 2 (FGF2). Tumor Biol. 2015;36(3):2127‐2134. 10.1007/s13277-014-2822-z. 25403884

[10] Wang J , Xu G , Shen F , Kang Y . miR‐132 targeting cyclin E1 suppresses cell proliferation in osteosarcoma cells. Tumor Biol. 2014;35(5):4859‐4865. 10.1007/s13277-014-1637-2. 24449507

[11] Joo MW , Shin SH , Kang YK , et al. Osteosarcoma in Asian populations over the age of 40 years: a multicenter study. Ann Surg Oncol. 2015;22(11):3557‐3564. 10.1245/s10434-015-4414-6 25676843

[12] Parry MC , Laitinen M , Albergo J , et al. Osteosarcoma of the pelvis. Bone Joint J. 2016;98B(4):555‐563. 10.1302/0301-620X.98B3.36583 27037440

[13] Liu CL , Tang YW , Li M , et al. Clinical characteristics and prognoses of six patients with multicentric giant cell tumor of the bone. Oncotarget. 2016;7(50):83795‐83805. 10.18632/oncotarget.13057 27823978 PMC5347806

[14] Cox TR , Erler JT , Rumney RMH . Established models and new paradigms for hypoxia‐driven cancer‐associated bone disease. Calcif Tissue Int. 2018;102(2):163‐173. 10.1007/s00223-017-0352-6 29098360 PMC5805797

[15] Martin E , Senders JT , ter Wengel PV , Smith TR , Broekman MLD . Treatment and survival of osteosarcoma and Ewing sarcoma of the skull: a SEER database analysis. Acta Neurochir. 2019;161(2):317‐325. 10.1007/s00701-018-3754-y 30578430 PMC6373276

[16] Green D , Mohorianu I , McNamara I , Dalmay T , Fraser WD . miR‐16 is highly expressed in Paget's associated osteosarcoma. Endocr Relat Cancer. 2017;24(5):L27‐L31. 10.1530/Erc-16-0487 28377382

[17] Li Z , Shen JX , Chan MTV , Wu WKK . The long non‐coding RNA SPRY4‐IT1: an emerging player in tumorigenesis and osteosarcoma. Cell Prolif. 2018;51(4): 10.1111/cpr.12446 PMC6528852 29484753

[18] Song B , Wang Y , Titmus MA , et al. Molecular mechanism of chemoresistance by miR‐215 in osteosarcoma and colon cancer cells. Mol Cancer. 2010;9(1):96 10.1186/1476-4598-9-96 20433742 PMC2881118

[19] Shen P , Cheng YF . Long noncoding RNA lncARSR confers resistance to Adriamycin and promotes osteosarcoma progression. Cell Death Dis. 2020;11(5):Article number: 362. 10.1038/S41419-020-2573-2. PMC7220921 32404870

[20] Jiang WX , Xia J , Xie SD , et al. Long non‐coding RNAs as a determinant of cancer drug resistance: towards the overcoming of chemoresistance via modulation of lncRNAs. Drug Resist Update. 2020;50:100683 10.1016/J.Drup.2020.100683 32146422

[21] Inkol JM , Poon AC , Mutsaers AJ . Inhibition of copper chaperones sensitizes human and canine osteosarcoma cells to carboplatin chemotherapy. Vet Comp Oncol. 2020; 10.1111/vco.12579 32060984

[22] Fanelli M , Tavanti E , Patrizio MP , et al. Cisplatin resistance in osteosarcoma: in vitro validation of candidate DNA repair‐related therapeutic targets and drugs for tailored treatments. Front Oncol. 2020;10:331 10.3389/Fonc.2020.00331 32211337 PMC7077033

[23] Zhang B , Zhang Y , Li RZ , Li JZ , Lu XC , Zhang Y . The efficacy and safety comparison of first‐line chemotherapeutic agents (high‐dose methotrexate, doxorubicin, cisplatin, and ifosfamide) for osteosarcoma: a network meta‐analysis. J Orthop Surg Res. 2020;15(1):51 10.1186/s13018-020-1576-0 32054494 PMC7020590

[24] Chen K , Jiao JB , Xue JW , et al. Ginsenoside CK induces apoptosis and suppresses proliferation and invasion of human osteosarcoma cells through the PI3K/mTOR/p70S6K1 pathway. Oncol Rep. 2020;43(3):886‐896. 10.3892/or.2020.7460 32020217 PMC7041301

[25] Rainusso N , Wang LL , Yustein JT . The adolescent and young adult with cancer: state of the art – bone tumors. Curr Oncol Rep. 2013;15(4):296‐307. 10.1007/s11912-013-0321-9 23690089

[26] Yan TQ , Wunder JS , Gokgoz N , et al. COPS3 amplification and clinical outcome in osteosarcoma. Cancer. 2007;109(9):1870‐1876. 10.1002/cncr.22595 17366602

[27] Zhang F , Yan TQ , Guo W , et al. Novel oncogene COPS3 interacts with Beclin1 and Raf‐1 to regulate metastasis of osteosarcoma through autophagy. J Exp Clin Canc Res. 2018;37:135 10.1186/S13046-018-0791-6 PMC6029018 29970115

[28] van Dartel M , Hulsebos TJM . Amplification and overexpression of genes in 17p11.2 similar to p12 in osteosarcoma. Cancer Genet Cytogenet. 2004;153(1):77‐80. 10.1016/j.cancergencyto.2004.03.007 15325100

[29] He ML , Wu Y , Zhao JM , Wang Z , Chen YB . AKT gene polymorphisms in susceptibility to osteosarcoma in a Chinese population. Asian Pac J Cancer Prev. 2013;14(9):5117‐5122. 10.7314/APJCP.2013.14.9.5117 24175786

[30] Gobin B , Baud'Huin M , Lamoureux F , et al. BYL719, a new alpha‐specific PI3K inhibitor: Single administration and in combination with conventional chemotherapy for the treatment of osteosarcoma. Int J Cancer. 2015;136(4):784‐796. 10.1002/ijc.29040 24961790

[31] Gao JJ , Ma S , Yang F , et al. miR‐193b exhibits mutual interaction with MYC, and suppresses growth and metastasis of osteosarcoma. Oncol Rep. 2020;44(1):139‐155. 10.3892/or.2020.7601 32377743 PMC7254955

[32] Samsa WE , Mamidi MK , Bashur LA , et al. The crucial p53‐dependent oncogenic role of JAB1 in osteosarcoma in vivo. Oncogene. 2020;39(23):4581‐4591. 10.1038/s41388-020-1320-6 32390003 PMC7274902

[33] Cabrera‐Andrade A , Lopez‐Cortes A , Jaramillo‐Koupermann G , et al. Gene prioritization through consensus strategy, enrichment methodologies analysis, and networking for osteosarcoma pathogenesis. Int J Mol Sci. 2020;21(3):1053 10.3390/Ijms21031053 PMC7038221 32033398

[34] Folk WP , Homlar K , Sakamuro D . PD‐0332991/palbociclib facilitates the physical interaction between RB1 and BIN1 to increase the vulnerability of pediatric osteosarcoma cells to PARP inhibition. Can Res. 2018;78(19). 10.1158/1538-7445.PEDCA17-B34

[35] Smolle MA , Heitzer E , Geigl JB , et al. A novel mutation in ATRX associated with intellectual disability, syndromic features, and osteosarcoma. Pediatr Blood Cancer. 2017;64(10): 10.1002/pbc.26522 28371197

[36] Yost KE , Soper SFC , Walker RL , et al. Rapid and reversible suppression of ALT by DAXX in osteosarcoma cells. Sci Rep. 2019;9(1):4544 10.1038/S41598-019-41058-8 30872698 PMC6418139

[37] Dhir A , Li R , Li GL , Dean J , Robin NH , Alva E . Simultaneous osteosarcoma and renal cell carcinoma with BRCA1 mutation in a young male adult with prior oligodendroglioma. Pediatr Blood Cancer. 2020;67(3): 10.1002/pbc.28116 31850619

[38] Tian ZZ , Guo XJ , Zhao YM , Fang Y . Decreased expression of long non‐coding RNA MEG3 acts as a potential predictor biomarker in progression and poor prognosis of osteosarcoma. Int J Clin Exp Pathol. 2015;8(11):15138‐15142. 26823857 PMC4713643

[39] Zhang Y , Duan G , Feng S . MicroRNA‐301a modulates doxorubicin resistance in osteosarcoma cells by targeting AMP‐activated protein kinase alpha 1. Biochem Biophys Res Commun. 2015;459(3):367‐373. 10.1016/j.bbrc.2015.02.101. 25727016

[40] Zhou C , Tan W , Lv H , Gao F , Sun J . Hypoxia‐inducible microRNA‐488 regulates apoptosis by targeting Bim in osteosarcoma. Cell Oncol (Dordr). 2016;39(5):463‐471. 10.1007/s13402-016-0288-2 27376839 PMC13001872

[41] Silva G , Aboussekhra A . p16(INK4A) inhibits the pro‐metastatic potentials of osteosarcoma cells through targeting the ERK pathway and TGF‐beta1. Mol Carcinog. 2016;55(5):525‐536. 10.1002/mc.22299 25728247

[42] Xing B , Ren C . Tumor‐suppressive miR‐99a inhibits cell proliferation via targeting of TNFAIP8 in osteosarcoma cells. Am J Transl Res. 2016;8(2):1082‐1090. 27158394 PMC4846951

[43] Wang Y , Shi S , Zhang Q , Dong H , Zhang J . MicroRNA‐206 upregulation relieves circTCF25‐induced osteosarcoma cell proliferation and migration. J Cell Physiol. 2020; 10.1002/jcp.29570

[44] Li S , Liu F , Pei Y , Wang W , Zheng K , Zhang X . Long noncoding RNA enhances the malignant characteristics of osteosarcoma by acting as a competing endogenous RNA on microRNA‐376a thereby upregulating dickkopf‐1. Aging. 2019;11(18):7678‐7693. 31525734 10.18632/aging.102280 PMC6781980

[45] Yu D , Xu X , Li S , Zhang K . LINC00514 drives osteosarcoma progression through sponging microRNA‐708 and consequently increases URGCP expression. Aging. 2020;12(8):6793‐6807. 10.18632/aging.103043 32325430 PMC7202513

[46] Gui D , Cao H . Long non‐coding RNA CDKN2B‐AS1 promotes osteosarcoma by increasing the expression of MAP3K3 via sponging miR‐4458. In Vitro Cell Dev Biol Anim. 2020;56(1):24‐33. 10.1007/s11626-019-00415-7 31950433

[47] Zhang PF , Gao C , Huang XY , et al. Cancer cell‐derived exosomal circUHRF1 induces natural killer cell exhaustion and may cause resistance to anti‐PD1 therapy in hepatocellular carcinoma. Mol Cancer. 2020;19(1):110 10.1186/s12943-020-01222-5 32593303 PMC7320583

[48] Lu Q , Liu TY , Feng HJ , et al. Circular RNA circSLC8A1 acts as a sponge of miR‐130b/miR‐494 in suppressing bladder cancer progression via regulating PTEN. Mol Cancer. 2019;18(1):111 10.1186/S12943-019-1040-0 31228937 PMC6588875

[49] Huang XX , Li Z , Zhang Q , et al. Circular RNA AKT3 upregulates PIK3R1 to enhance cisplatin resistance in gastric cancer via miR‐198 suppression. Mol Cancer. 2019;18(1):71 10.1186/s12943-019-0969-3 30927924 PMC6441201

[50] Wu K , Liao X , Gong YL , et al. Circular RNA F‐circSR derived from SLC34A2‐ROS1 fusion gene promotes cell migration in non‐small cell lung cancer. Mol Cancer. 2019;18(1):98 10.1186/s12943-019-1028-9 31118036 PMC6530145

[51] Ding LX , Zhao YY , Dang SW , et al. Circular RNA circ‐DONSON facilitates gastric cancer growth and invasion via NURF complex dependent activation o transcription factor SOX4. Mol Cancer. 2019;18(1):45 10.1186/s12943-019-1006-2 30922402 PMC6437893

[52] Wang SH , Zhang YJ , Cai Q , et al. Circular RNA FOXP1 promotes tumor progression and Warburg effect in gallbladder cancer by regulating PKLR expression. Mol Cancer. 2019;18(1):145 10.1186/S12943-019-1078-Z 31623628 PMC6796492

[53] Zhang QG , Wang WW , Zhou QB , et al. Roles of circRNAs in the tumour microenvironment. Mol Cancer. 2020;19(1):14 10.1186/s12943-019-1125-9 31973726 PMC6977266

[54] Yang F , Hu AP , Li D , et al. Circ‐HuR suppresses HuR expression and gastric cancer progression by inhibiting CNBP transactivation. Mol Cancer. 2019;18(1):158 10.1186/S12943-019-1094-Z 31718709 PMC6852727

[55] Kong Y , Li MT , Luo YM , et al. circNFIB1 inhibits lymphangiogenesis and lymphatic metastasis via the miR‐486‐5p/PIK3R1/VEGF‐C axis in pancreatic cancer. Mol Cancer. 2020;19(1):82 10.1186/S12943-020-01205-6 32366257 PMC7197141

[56] He JH , Huang ZY , He ML , et al. Circular RNA MAPK4 (circ‐MAPK4) inhibits cell apoptosis via MAPK signaling pathway by sponging miR‐125a‐3p in gliomas. Mol Cancer. 2020;19(1):17 10.1186/s12943-019-1120-1 31992303 PMC6986105

[57] Jie MM , Wu YR , Gao MY , et al. CircMRPS35 suppresses gastric cancer progression via recruiting KAT7 to govern histone modification. Mol Cancer. 2020;19(1):56 10.1186/S12943-020-01160-2 32164722 PMC7066857

[58] Liu P , Li X , Guo X , et al. Circular RNA DOCK1 promotes bladder carcinoma progression via modulating circDOCK1/hsa‐miR‐132‐3p/Sox5 signalling pathway. Cell Prolif. 2019;52(4):e12614 10.1111/cpr.12614 30983072 PMC6668968

[59] Wei Y , Zhang Y , Meng Q , Cui L , Chang X . Hypoxia‐induced circular RNA has_circRNA_403658 promotes bladder cancer cell growth through activation of LDHA. Am J Transl Res. 2019;11(11):6838‐6849. 31814891 PMC6895513

[60] Tian F , Yu C , Wu M , Wu X , Wan L , Zhu X . MicroRNA‐191 promotes hepatocellular carcinoma cell proliferation by has_circ_0000204/miR‐191/KLF6 axis. Cell Prolif. 2019;52(5):e12635. 31334580 10.1111/cpr.12635 PMC6797514

[61] Huang X , He M , Huang S , et al. Circular RNA circERBB2 promotes gallbladder cancer progression by regulating PA2G4‐dependent rDNA transcription. Mol Cancer. 2019;18(1):166 10.1186/S12943-019-1098-8 31752867 PMC6868820

[62] Li JF , Huang CC , Zou YF , Ye J , Yu J , Gui YT . CircTLK1 promotes the proliferation and metastasis of renal cell carcinoma by sponging miR‐136‐5p. Mol Cancer. 2020;19(1):103 10.1186/s12943-020-01225-2 32503552 PMC7275467

[63] Soghli N , Qujeq D , Yousefi T , Soghli N . The regulatory functions of circular RNAs in osteosarcoma. Genomics. 2020;112(4):2845‐2856. 10.1016/j.ygeno.2020.03.024 32243895

[64] Wang CC , Jing JH , Cheng L . Emerging roles of non‐coding RNAs in the pathogenesis, diagnosis and prognosis of osteosarcoma. Invest New Drug. 2018;36(6):1116‐1132. 10.1007/s10637-018-0624-7 30079443

[65] Liu WH , Zhang JJ , Zou CY , et al. Microarray expression profile and functional analysis of circular RNAs in Osteosarcoma. Cell Physiol Biochem. 2017;43(3):969‐985. 10.1159/000481650 28957794

[66] Zheng SN , Qian ZY , Jiang F , et al. CircRNA LRP6 promotes the development of osteosarcoma via negatively regulating KLF2 and APC levels. Am J Transl Res. 2019;11(7):4126‐4138. 31396323 PMC6684910

[67] Zhao YB , Zhang J . CircSAMD4A accelerates cell proliferation of osteosarcoma by sponging miR‐1244 and regulating MDM2 mRNA expression. Biochem Biophys Res Comm. 2019;516(1):102‐111. 10.1016/j.bbrc.2019.05.182 31200957

[68] Zhang Y , Li JL , Wang YS , Jing JH , Li J . The roles of circular RNAs in osteosarcoma. Med Sci Monitor. 2019;25:6378‐6382. 10.12659/Msm.915559 PMC6724563 31446435

[69] Vo J , Cieslik M , Zhang Y , et al. The landscape of circular RNA in Cancer. Cell. 2019;176(4):869‐81.e13. 10.1016/j.cell.2018.12.021 30735636 PMC6601354

[70] Yang Y , Fan X , Mao M , et al. Extensive translation of circular RNAs driven by N‐methyladenosine. Cell Res. 2017;27(5):626‐641. 10.1038/cr.2017.31 28281539 PMC5520850

[71] You X , Vlatkovic I , Babic A , et al. Neural circular RNAs are derived from synaptic genes and regulated by development and plasticity. Nat Neurosci. 2015;18(4):603‐610. 10.1038/nn.3975 25714049 PMC4376664

[72] Chen Y , Chen C , Mai T , et al. Genome‐wide, integrative analysis of circular RNA dysregulation and the corresponding circular RNA‐microRNA‐mRNA regulatory axes in autism. Genome Res. 2020;30(3):375‐391. 10.1101/gr.255463.119 32127416 PMC7111521

[73] Xi YZ , Fowdur M , Liu Y , Wu H , He ML , Zhao JM . Differential expression and bioinformatics analysis of circRNA in osteosarcoma. Biosci Rep. 2019;39: 10.1042/Bsr20181514 PMC6522716 31036604

[74] Ji X , Shan L , Shen P , He M . Circular RNA circ_001621 promotes osteosarcoma cells proliferation and migration by sponging miR‐578 and regulating VEGF expression. Cell Death Dis. 2020;11(1):18 10.1038/s41419-019-2204-y 31907361 PMC6944700

[75] Qiu Y , Pu C , Li YC , Qi BC . Construction of a circRNA‐miRNA‐mRNA network based on competitive endogenous RNA reveals the function of circRNAs in osteosarcoma. Cancer Cell Int. 2020;20:48 10.1186/s12935-020-1134-1 32063749 PMC7011443

[76] Zhu KP , Ma XL , Zhang L , et al. Screening circular RNA related to chemotherapeutic resistance in osteosarcoma by RNA sequencing. Epigenomics. 2018;10(10):1327‐1346. 10.2217/epi-2018-0023 30191736

[77] Zhong Z , Lv M , Chen J . Screening differential circular RNA expression profiles reveals the regulatory role of circTCF25‐miR‐103a‐3p/miR‐107‐CDK6 pathway in bladder carcinoma. Sci Rep. 2016;6:30919 10.1038/srep30919 27484176 PMC4971518

[78] Wang Y , Shi S , Zhang Q , Dong H , Zhang J . MicroRNA‐206 upregulation relieves circTCF25‐induced osteosarcoma cell proliferation and migration. J Cell Physiol. 2020; 10.1002/jcp.29570

[79] Zheng F , Xu R . CircPVT1 contributes to chemotherapy resistance of lung adenocarcinoma through miR‐145‐5p/ABCC1 axis. Biomed Pharmacother. 2020;124:109828 10.1016/j.biopha.2020.109828 31986409

[80] Liu YP , Wan J , Long F , Tian J , Zhang C . circPVT1 Facilitates invasion and metastasis by regulating miR‐205‐5p/c‐FLIP Axis in osteosarcoma. Cancer Manag Res. 2020;12:1229‐1240. 10.2147/Cmar.S231872 32110097 PMC7035890

[81] Yan M , Gao H , Lv Z , et al. Circular RNA PVT1 promotes metastasis via regulating of miR‐526b/FOXC2 signals in OS cells. J Cell Mol Med. 2020;24(10):5593‐5604. 10.1111/jcmm.15215 32249539 PMC7214167

[82] Wang R , Zhang S , Chen X , et al. EIF4A3‐induced circular RNA MMP9 (circMMP9) acts as a sponge of miR‐124 and promotes glioblastoma multiforme cell tumorigenesis. Mol Cancer. 2018;17(1):166 10.1186/s12943-018-0911-0 30470262 PMC6260852

[83] Pan GJ , Hu T , Chen XW , Zhang C . Upregulation of circMMP9 promotes osteosarcoma progression via targeting miR‐1265/CHI3L1 Axis. Cancer Manag Res. 2019;11:9225‐9231. 10.2147/Cmar.S226264 31754311 PMC6825504

[84] Zhang Z , Pu F , Wang B , Wu Q , Liu J , Shao Z . Hsa_circ_0000285 functions as a competitive endogenous RNA to promote osteosarcoma progression by sponging hsa‐miRNA‐599. Gene Ther. 2020;27(5):186‐195. 10.1038/s41434-019-0112-5 31784675

[85] Verrecchia F , Rédini F . Transforming growth factor‐β signaling plays a pivotal role in the interplay between osteosarcoma cells and their microenvironment. Front Oncol. 2018;8:133 10.3389/fonc.2018.00133 29761075 PMC5937053

[86] Wang L , Wang P , Su X , Zhao B . Circ_0001658 promotes the proliferation and metastasis of osteosarcoma cells via regulating miR‐382‐5p/YB‐1 axis. Cell Biochem Funct. 2020;38(1):77‐86. 10.1002/cbf.3452 31758574

[87] Jin Y , Li L , Zhu T , Liu G . Circular RNA circ_0102049 promotes cell progression as ceRNA to target MDM2 via sponging miR‐1304‐5p in osteosarcoma. Pathol Res Pract. 2019;215(12):152688 10.1016/j.prp.2019.152688 31727503

[88] Tan X , Tan D , Li H , Lin Y , Wen Z , Zeng C . circEPSTI1 Acts as a ceRNA to Regulate the Progression of Osteosarcoma. Curr Cancer Drug Targets. 2020;20(4):288‐294. 10.2174/1568009619666191107140948 31702512

[89] Cao J , Liu X . Circular RNA 0060428 sponges miR‐375 to promote osteosarcoma cell proliferation by upregulating the expression of RPBJ. Gene. 2020;740:144520 10.1016/j.gene.2020.144520 32130980

[90] Du Y , Guo L , Pan H , Liang Y , Li X . Circ_ANKIB1 stabilizes the regulation of miR‐19b on SOCS3/STAT3 pathway to promote osteosarcoma cell growth and invasion. Hum Cell. 2020;33(1):252‐260. 10.1007/s13577-019-00298-6 31667786

[91] Chen J , Liu G , Wu Y , et al. CircMYO10 promotes osteosarcoma progression by regulating miR‐370‐3p/RUVBL1 axis to enhance the transcriptional activity of β‐catenin/LEF1 complex via effects on chromatin remodeling. Mol Cancer. 2019;18(1):150 10.1186/s12943-019-1076-1 31665067 PMC6819556

[92] Zheng S , Qian Z , Jiang F , et al. CircRNA LRP6 promotes the development of osteosarcoma negatively regulating KLF2 and APC levels. Am J Transl Res. 2019;11(7):4126‐4138. 31396323 PMC6684910

[93] Li S , Pei Y , Wang W , Liu F , Zheng K , Zhang X . Circular RNA 0001785 regulates the pathogenesis of osteosarcoma as a ceRNA by sponging miR‐1200 to upregulate HOXB2. Cell Cycle. 2019;18(11):1281‐1291. 10.1080/15384101.2019.1618127 31116090 PMC6592237

[94] Li X , Sun X , Xu H , Pan H , Liu Y , He L . Circ_ORC2 enhances the regulatory effect of miR‐19a on its target gene PTEN to affect osteosarcoma cell growth. Biochem Biophys Res Comm. 2019;514(4):1172‐1178. 10.1016/j.bbrc.2019.04.188 31103262

[95] Jin J , Chen A , Qiu W , et al. Dysregulated circRNA_100876 suppresses proliferation of osteosarcoma cancer cells by targeting microRNA‐136. J Cell Biochem. 2019;120(9):15678‐15687. 10.1002/jcb.28837 31069828

[96] Wu Y , Xie Z , Chen J , et al. Circular RNA circTADA2A promotes osteosarcoma progression and metastasis by sponging miR‐203a‐3p and regulating CREB3 expression. Mol Cancer. 2019;18(1):73 10.1186/s12943-019-1007-1 30940151 PMC6444890

[97] Zhu K , Niu L , Wang J , et al. Circular RNA hsa_circ_0000885 levels are increased in tissue and serum samples from patients with osteosarcoma. Med Sci Monitor. 2019;25:1499‐1505. 10.12659/msm.914899 PMC6400018 30802235

[98] Wang L , Zhang G , Kang F , Zhang L , Zhang Y . hsa_circ0021347 as a potential target regulated by B7–H3 in modulating the malignant characteristics of osteosarcoma. Biomed Res Int. 2019;2019:9301989 10.1155/2019/9301989 31950059 PMC6948356

[99] Li S , Pei Y , Wang W , Liu F , Zheng K , Zhang X . Extracellular nanovesicles‐transmitted circular RNA has_circ_0000190 suppresses osteosarcoma progression. J Cell Mol Med. 2020;24(3):2202‐2214. 10.1111/jcmm.14877 31923350 PMC7011131

[100] Hu Y , Gu J , Shen H , et al. Circular RNA LARP4 correlates with decreased Enneking stage, better histological response, and prolonged survival profiles, and it elevates chemosensitivity to cisplatin and doxorubicin via sponging microRNA‐424 in osteosarcoma. J Clin Lab Anal. 2020;34(2):e23045 10.1002/jcla.23045 31642110 PMC7031593

[101] Li F , Zhang L , Li W , et al. Circular RNA ITCH has inhibitory effect on ESCC by suppressing the Wnt/β‐catenin pathway. Oncotarget. 2015;6(8):6001‐6013. 10.18632/oncotarget.3469 25749389 PMC4467417

[102] Huang G , Zhu H , Shi Y , Wu W , Cai H , Chen X . cir‐ITCH plays an inhibitory role in colorectal cancer by regulating the Wnt/β‐catenin pathway. PLoS One. 2015;10(6):e0131225 10.1371/journal.pone.0131225 26110611 PMC4482251

[103] Wan L , Zhang L , Fan K , Cheng Z , Sun Q , Wang J . Circular RNA‐ITCH suppresses lung cancer proliferation via inhibiting the Wnt/β‐catenin pathway. Biomed Res Int. 2016;2016:1579490 10.1155/2016/1579490 27642589 PMC5013215

[104] Li H , Lan M , Liao X , Tang Z , Yang C . cir‐ITCHCircular RNA promotes osteosarcoma migration and invasion through /miR‐7/EGFR Pathway. Technol Cancer Res Treat. 2020;19:1533033819898728 10.1177/1533033819898728 31960764 PMC6974758

[105] Ren C , Liu J , Zheng B , Yan P , Sun Y , Yue B . The circular RNA circ‐ITCH acts as a tumour suppressor in osteosarcoma via regulating miR‐22. Artif Cells Nanomed Biotechnol. 2019;47(1):3359‐3367. 10.1080/21691401.2019.1649273 31387405

